# HMB45/PRAME, a Novel Double Staining for the Diagnosis of Melanocytic Neoplasms: Technical Aspects, Results, and Comparison With Other Commercially Available Staining (PRAME and Melan A/PRAME)

**DOI:** 10.1097/PAI.0000000000000972

**Published:** 2021-09-10

**Authors:** Marco Grillini, Costantino Ricci, Vincenzo Pino, Silvia Pedrini, Michelangelo Fiorentino, Barbara Corti

**Affiliations:** * Pathology Unit, IRCSS Sant’Orsola-Malpighi Hospital and University of Bologna; † Pathology Unit, Maggiore Hospital; ‡ Department of Experimental, Diagnostic, and Specialty Medicine (DIMES), University of Bologna, Bologna, Italy

**Keywords:** melanoma, immunohistochemistry, HMB45, PRAME, HMB45/PRAME

## Abstract

PRAME (*PR*eferentially expressed *A*ntigen in *ME*lanoma) is a tumor-associated antigen that was recently found to be expressed by malignant melanocytic lesions but not by benign ones, thus resulting useful in this diagnostic field. PRAME could also be expressed by some normal tissues and nonmelanocytic tumors, suggesting as caution should be adopted to use PRAME as a “pan-melanoma” marker for the differential diagnosis with other malignant tumors. Until now, PRAME expression was exclusively investigated through single staining with a monoclonal antibody targeting PRAME and with double staining for Melan A/PRAME found to be useful in specific diagnostic sets. Herein, we studied the expression of PRAME in 40 melanocytic lesions and 23 nonmelanocytic ones using PRAME, Melan A/PRAME, and novel double staining for HMB45/PRAME. Although our results need to be validated, they support the adoption of HMB45/PRAME, alone or in combination with PRAME and Melan A/PRAME, as a helpful marker in the diagnosis of melanocytic neoplasms with a high concordance rate between primary melanoma and corresponding metastases.

PRAME (*PR*eferentially expressed *A*ntigen in *ME*lanoma) is a tumor-associated antigen that was firstly identified by autologous T cells in patients with metastatic melanoma (M).^1^ Recently, Lezcano et al^2^ showed as immunohistochemical (IHC) assessment of PRAME could be extraordinarily useful in the differential diagnosis of melanocytic lesions, being expressed in the majority of M but not in nevi (N). Based on these encouraging results, PRAME has been subsequently tested in numerous and different fields of melanocytic pathology [uveal M, halo N, atypical Spitz tumor, nodal nevi (NN) vs. melanoma metastasis (MM)] always showing to be helpful.^2^–^11^ Besides, PRAME was investigated in other fields of human pathology and found to be expressed in some normal tissues (testis, ovary, placenta, adrenal gland, endometrium, and lymphocytes subclasses) and nonmelanocytic tumors (sarcomas, embryonal carcinoma, leukemias, and lymphomas).^12^–^20^ These findings highlighted as caution is needed in the adoption of PRAME as a “pan-melanoma” diagnostic marker, especially in incisional and superficial biopsies or in the setting of metastatic disease by the unknown primary tumor.^5^,^6^,^8^,^12^,^14^,^15^,^18^,^19^ Until now, the IHC for PRAME was exclusively based on single staining (SS) with monoclonal antibody (mAb EPR20330, #219650; Abcam) commercially available for automated Leica-Bond stainer platform.^2^–^19^ Only Lezcano et al^5^ tested double staining (DS) for Melan A/PRAME in a small subset of NN (16 cases) and found as this DS could be useful in evaluating PRAME only in melanocytic cells, helping to differentiate them from the PRAME (+) inflammatory cells/lymphocytes resident in the lymph nodes and from the rare but possible lymph node localizations of other PRAME (+) tumors. In the present study, we firstly tested a DS for HMB45/PRAME in a small cohort of melanocytic lesions, providing the technical and methodological aspects in comparison with other available SS (PRAME, HMB45, and Melan A) and DS (Melan A/PRAME), analyzing the results and discussing its potential implications in a diagnostic routine set.

## MATERIALS AND METHODS

We retrospectively analyzed 40 melanocytic and 23 nonmelanocytic cases diagnosed between January 1, 2021, and March 1, 2021, at the Pathology Unit, IRCCS Azienda Ospedaliero-Universitaria di Bologna, Policlinico di Sant’Orsola. Routine histologic analysis was performed on formalin-fixed, paraffin-embedded, 3 μm thick sections and stained with hematoxylin and eosin. All the cases were evaluated with SS (Melan A, HMB45, and PRAME) and DS (Melan A/PRAME and HMB45/PRAME). IHC was performed on 3 μm thick sections of formalin-fixed, paraffin-embedded tissue on the BenchMark ULTRA automated immunostainer (Ventana Medical Systems-Roche Diagnostics, Switzerland). In both DS, PRAME nuclear staining was visualized with DAB (brown chromogen-OptiView DAB Detection Kit; Ventana), whereas Melan A and HMB45 cytoplasmatic staining were visualized with FastRed (red chromogen-ultraView Red Detection Kit; Ventana) to minimize difficulties in interpretation due to cytoplasmic melanin pigment. To enhance PRAME brown signal in the DS, a further step with an amplification kit was added (OptiView Amplification Kit; Ventana). IHC protocols, clone antibodies, and other technical data are summarized in Table 1. PRAME immunopositivity was defined as any immunolabeling within the tumor, as previously described by Lezcano et al.^1^ All the slides (hematoxylin and eosin, SS, and DS) were assessed by 2 expert dermatopathologists (B.C. and C.R.) to grade PRAME based on the percentage of immunoreactive tumor cells, as previously described by Lezcano et al (0: 0%; 1+: 1% to 25%; 2+: 26% to 50%; 3+: 51% to 75%; 4+: ≥76%) and to evaluate cytoplasmatic coexpression of Melan A and HMB45.^1^ Histologic diagnosis and IHC data are summarized in Table 2.

**TABLE 1:** Immunohistochemical Protocols, Clones, Dilutions, and Companies of the Antibodies Used in the Study

Antibody	Species	Clone	Dilution	Catalog Number	Source
Anti-Melan A/MART-1	Mouse	A103	RTU	790-2990	Ventana
Anti-HMB45	Mouse	HMB45	RTU	790-4366	Ventana
Anti-PRAME	Rabbit	EPR20330	1:5000	ab219650	Abcam, UK
Protocol name	Antigen retrieval	Antibody incubation	Visualization		
PRAME (SS)	Ultra CC1×32 min at 95°C	20 min at 36°C	OptiView DAB Detection Kit		
Melan A/PRAME (DS)	Ultra CC1×32 min at 95°C	24 min at RT (Melan A)	ultraView Universal Alkaline Phosphatase Red Detection Kit+Amplification Kit (Melan A)		
		24 min at 36°C (PRAME)	OptiView DAB Detection Kit+OptiView Amplification Kit (PRAME)		
HMB45/PRAME (DS)	Ultra CC1×32 min at 95°C	20 min at RT (HMB45)	ultraView Universal Alkaline Phosphatase Red Detection Kit (HMB45)		
		20 min at 36°C (PRAME)	OptiView DAB Detection Kit+OptiView Amplification Kit (PRAME)		

DAB indicates 3,3′-diaminobenzidine; DS, double staining; HMB45, *H*uman *M*elanoma *B*lack *45*; M, melanoma; Melan A/MART-1 *Melan*oma *A*ntigen/*M*elanoma *A*ntigen *R*ecognized by *T* cells *1*; PRAME, *PR*eferentially expressed *A*ntigen in *ME*lanoma; RT, room temperature; RTU, ready to use; SS, single staining.

**TABLE 2:** Histologic Diagnosis and Immunohistochemical Data of the Cases Series

Patient Number	Case Number	Histologic Diagnosis	PRAME	Melan A/MART-1	MHB45
1	1	MN	4+	+ (d; w)	+ (f; s)
2	2	LMM	4+	+ (d; w)	+ (d; s)
3	3	MM (NM)	4+	+ (d; w)	+ (d; s)
	4	MM (NM)	4+	+ (d; w)	+ (d; m)
	5	MM (NM)	4+	+ (d; w)	+ (d; s)
	6	MM (NM)	4+	+ (d; m)	+ (d; m)
	7	MM (NM)	4+	+ (d; m)	+ (d; s)
	8	MM (NM)	4+	+ (d; w)	+ (d; s)
4	9	MN	3+	+ (d; m)	−
5	10	MM (MIT)	4+	+ (d; w)	+ (d; s)
	11	MM (MIT)	3+	+ (d; w)	+ (d; s)
	12	MM (NM)	4+	+ (d; w)	+ (d; s)
	13	MM (NM)	4+	+ (d; w)	+ (d; s)
	14	MA	4+	+ (d; m)	+ (d; s)
6	15	SSM	4+	+ (d; w)	+ (d; s)
	16	MM (NM)	4+	+ (d; w)	+ (d; s)
7	17	MN	4+	−	+ (i; s)
8	18	SSM	4+	+ (d; w)	+ (d; s)
9	19	MN	1+	+ (d; m)	−
10	20	LMM	4+	+ (d; w)	+ (d; s)
11	21	MA	4+	+ (d; w)	+ (i; s)
12	22	SSM	4+	+ (d; w)	+ (d; s)
	23	DN	1+	+ (d; w)	+ (d; w)
13	24	DN	1+	+ (d; w)	+ (d; w)
	25	N	0	+ (d; w)	−
14	26	NN	0	+ (d; w)	−
	27	NN	0	+ (d; w)	−
	28	DN	0	+ (d; m)	+ (i; s)
15	29	NN	0	+ (d; w)	−
	30	NN	0	+ (d; w)	−
	31	NN	0	+ (d; w)	−
	32	NN	0	+ (d; m)	−
	33	SSM	0	+ (d; m)	+ (d; s)
16	34	DN	1+	+ (d; s)	+ (i; s)
17	35	SN	0	+ (d; m)	+ (f; s)
18	36	BN	0	+ (d; m)	+ (d; s)
19	37	HN	0	+ (d; m)	+ (d; s)
20	38	N	0	+ (d; w)	−
21	39	DN	1+	+ (d; m)	+ (d; s)
	40	DN	0	+ (d; m)	+ (i; s)
22	41	BCC	0	−	−
23	42	BCC	0	−	−
24	43	BCC	0	−	−
25	44	SCC	0	−	−
26	45	SCC	0	−	−
27	46	SCC	0	−	−
28	47	AFX	0	−	−
29	48	AFX	0	−	−
30	49	AFX	0	−	−
31	50	DPS	0	−	−
32	51	DPS	0	−	−
33	52	DPS	0	−	−
34	53	NMC	0	−	−
35	54	NMC	0	−	−
36	55	NMC	0	−	−
37	56	NMC	0	−	−
38	57	NMC	0	−	−
39	58	NMC	0	−	−
40	59	NMC	0	−	−
41	60	NMC	0	−	−
42	61	NMC	0	−	−
43	62	NMC	0	−	−
44	63	NMC	0	−	−
45	64	NMC	0	−	−

PRAME has been graded as previously described by Lezcano et al.^2^

− indicates negative; +, positive; AFX, atypical fibroxanthoma; BCC, basal cell carcinoma; d, diffuse stain (in terms of number/% of positive cells); DN, dysplastic nevus; DPS, dermal pleomorphic sarcoma; f, focal stain (in terms of number/% of positive cells); HMB45, *H*uman *M*elanoma *B*lack *45*; HN, halo nevus; i, intermediate stain (in terms of number/% of positive cells); LMM, lentigo maligna-melanoma; m, moderate (stain intensity, regardless of number/% of positive cells); MA, acral melanoma; Melan a/MART-1, *Melan*oma *a*ntigen/*M*elanoma *a*ntigen *R*ecognized by *T* cells *1*; MIT, in-transit metastasis; MM, melanoma metastasis; MN, nodal metastasis; MN, nodular melanoma; N, common nevus; NMC, lymph node metastases of poorly differentiated carcinoma (lung: 6, colon: 3, bladder: 2); NN, nodal nevus; PRAME, *PR*eferentially expressed *A*ntigen in *ME*lanoma; s, strong (stain intensity, regardless of number/% of positive cells); SCC, squamous cell carcinoma; SN, Spitz nevus; SSM, superficial spreading melanoma; w, weak (stain intensity, regardless of number/% of positive cells).

## RESULTS AND DISCUSSION

**FIGURE 1:**
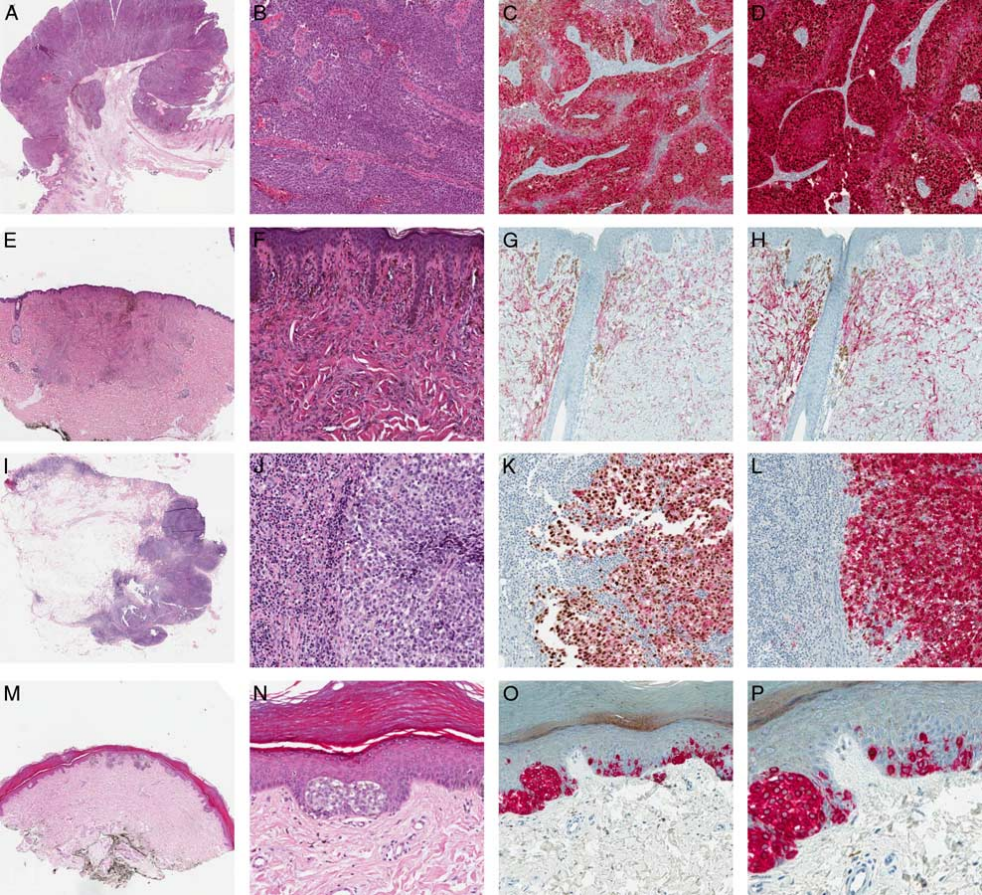
A–D, Case number 1 (Table 2): Nodular melanoma. A, H&E (original magnification, ×20). B, H&E (original magnification, ×200). C, DS for Melan A/PRAME (original magnification, ×200). D, DS for HMB45/PRAME (original magnification, ×200). The lesion showed diffuse nuclear staining for PRAME (4+) and cytoplasmatic 1 for both HMB45 and Melan A (this latter less intense). E–H, Case number 36 (Table 2): Blue nevus. E, H&E (original magnification, ×20). F, H&E (original magnification, ×200). G, DS for Melan A/PRAME (original magnification, ×200). H, DS for HMB45/PRAME (original magnification, ×200). The lesion showed diffuse cytoplasmatic staining for both HMB45 and Melan A (this latter less intense) but not nuclear one for PRAME (0). I–L, Case number 15 (Table 2): Nodal metastasis. I, H&E (original magnification, ×20). J, H&E (original magnification, ×200). K, DS for Melan A/PRAME (original magnification, ×200). L, DS for HMB45/PRAME (original magnification, ×200). The lesion showed diffuse nuclear staining for PRAME (4+) and cytoplasmatic one for both HMB45 and Melan A (this latter less intense). There are rare PRAME (+) inflammatory cells/lymphocytes resident in the lymph node, with no cytoplasmatic staining for both Melan A and HMB45. M–P, Case number 39 (Table 2): Dysplastic nevus (interdigital skin of the foot). M, H&E (original magnification, ×20). N, H&E (original magnification, ×200). O, DS for HMB45/PRAME (original magnification, ×200). P, DS for HMB45/PRAME (original magnification, ×400). The lesion showed strong and diffuse cytoplasmatic staining for HMB45 with rare cells positive for PRAME (1+). PRAME has been graded as previously described by Lezcano et al.^2^ DS indicates double staining; H&E, hematoxylin and eosin; HMB45, *H*uman *M*elanoma *B*lack *45*; Melan A/MART-1, *Melan*oma *A*ntigen/*M*elanoma *A*ntigen *R*ecognized by *T* cells *1*; PRAME, *PR*eferentially expressed *A*ntigen in *ME*lanoma.

Of the 23 M and MM tested, 21 (91.3%) were scored as 3+ (2/23, 8.7%) and/or 4+ (19/23, 82.6%). Conversely, 13/17 (76.5%) N and NN lacked any staining (0), and only 4/17 (23.5%) showed PRAME immunoreactivity in a minor population of melanocytes (1+). The nonmelanocytic tumors chosen as negative controls were completely negative (0) (Table 2). All the melanocytic lesions showed variable cytoplasmatic expression of Melan A and HMB45 depending on the analyzed sample (Table 2, Fig. 1). Notably, all the nodal metastases (NM) (9/9, 100%) were positive for HMB45 and Melan A (both diffuse), whereas all the NN (6/6, 100%) turned out positive for Melan A (diffuse) and negative for HMB45. In patients #3, #5, and #15, NN and NM were multiple (distinct multifocal lesions) and detected with the updated European Organisation for Research and Treatment of Cancer (EORTC) protocol for sentinel lymph node biopsy (SLNB).^21^ One M (patient #15) composed of atypical epithelioid cells [pagetoid spread, dermal mitosis, high Ki-67 index, HMB45 (+) and p16 (−)] was PRAME (−) and deserves special mention. The corresponding SLNB showed 4 distinct melanocytic deposits of small and bland cells [HMB45 and PRAME (−), p16 and Melan A (+), with a low Ki-67 index] diagnosed as multiple NN. Curiously, also Lezcano et al^2^ identified an analogous case [patient #17, with both M and NM PRAME (−)] in their case series. In all cutaneous samples, isolated PRAME (+) melanocytes and cytoplasmic labeling of sebaceous glands were noted in the adjacent normal skin; in the lymph nodes, rare PRAME (+) lymphocytes and/or immune cells were detected. These 2 findings are in line with what was previously described by other authors.^2^–^4^ Comparing the 2 DS, all cases positive for both HMB45 and Melan A showed more intense staining for HMB45 rather than Melan A, regardless of the percentage of positive cells (Table 2, Fig. 1); notably, Melan A showed more intense staining in SS than in DS, always regardless of the percentage of positive cells. In all cases, SS (PRAME, Melan A, and HMB45) showed results completely superimposable to DS, with no discrepancies in terms of nuclear and cytoplasmatic staining. Although on a small case series, our study supports PRAME as a helpful marker in the differential diagnosis between benign and malignant melanocytic neoplasms.^2^–^11^ In this study, we firstly tested a novel DS for HMB45/PRAME and found promising results, also compared with the other commercially available SS (PRAME) and DS (Melan A/PRAME). This latter has been tested only in a small subset of NN, and the authors concluded as the integration of Melan A in a DS was greatly useful for the correct evaluation of PRAME in the melanocytic cells of NN rather than in the background inflammatory cells/lymphocytes of the lymph node.^5^ Our results support this finding, highlighting as these 2 DS could not be interchangeable and are probably useful in the differential diagnosis between NN [Melan A (+), HMB45 and PRAME (−)] and MM [Melan A, HMB45, and PRAME (+)], especially adopting the updated EORTC protocol for SLNB. This protocol greatly increases the detection rate of NN and MM (also as distinct and synchronous lesions in the same SLNB sample), with the “lymph node metastatic burden” being important for prognostic stratification and therapeutic choice in these patients.^21^–^24^ For these reasons, the specular and integrable results provided by these 2 DS could be useful for the assessment of the lymph node metastatic burden in SLNB, especially in selected difficult cases. The other 2 noteworthy aspects of the present study are: (a) the high concordance of PRAME in cases with primary M and corresponding MM; (b) the possibility of using the DS in the diagnosis of metastatic disease by an unknown primary tumor (Table 2). The first data are in line with those found by previous studies and suggest to evaluate PRAME comparing multiple lesions of the same patients (M, NN, MM) in association with histology and additional S (HMB45, Ki-67, p16; patient #15). The second point shows as, although a diffuse positivity for PRAME (4+) could reasonably favor a diagnosis of M in the appropriate clinical set, rare cases of M and MM could be negative for PRAME (patient #15), as well as other tumors (sarcomas, carcinomas of the female genital tracts, leukemias and germ cell tumors) could be positive for PRAME.^12^–^20^ In this context, DS showing positivity for both PRAME and melanocytic markers (Melan A and HMB45) strongly encourages a diagnosis of M, especially on small/incisional biopsies with the risk that material could be consumed on serial sections. Last, DS showed more intense staining for HMB45 rather than Melan A, with Melan A being more intense in SS rather than in DS (Table 2, Fig. 1). However, in the only figure with DS (Melan A/PRAME) provided by Lezcano et al,^5^ the cytoplasmatic staining obtained with Melan A in SS and DS was comparable with ours, so leading us to suppose that these authors experienced the same technical issues. Nevertheless, future studies are needed to investigate these issues and verify whether these results may be affected by the samples, the procedures (incubation time, temperatures, detection kits), or the antibodies (clones, dilution). In conclusion, DS for HMB45/PRAME showed encouraging results for its potential application in the diagnosis of melanocytic neoplasms and the differential diagnosis between M and other malignant tumors, confirming and expanding the amount of data regarding PRAME. Future studies on larger case series are needed to validate these results and to improve some technical issues.
